# The trophoblast cell surface antigen 2 and miR-125b axis in urothelial bladder cancer

**DOI:** 10.18632/oncotarget.17407

**Published:** 2017-04-25

**Authors:** Chiara Avellini, Caterina Licini, Raffaella Lazzarini, Rosaria Gesuita, Emanuela Guerra, Giovanni Tossetta, Clara Castellucci, Stefano Raffaele Giannubilo, Antonio Procopio, Saverio Alberti, Roberta Mazzucchelli, Fabiola Olivieri, Daniela Marzioni

**Affiliations:** ^1^ Department of Experimental and Clinical Medicine, Università Politecnica delle Marche, Ancona, Italy; ^2^ Department of Clinical and Molecular Sciences, DISCLIMO, Università Politecnica delle Marche, Ancona, Italy; ^3^ Centre of Epidemiology, Biostatistics and Medical Information Technology, Università Politecnica delle Marche, Ancona, Italy; ^4^ Unit of Cancer Pathology, CeSI-MeT, “G. d'Annunzio” University, Chieti, Italy; ^5^ ONCOXX Biotech SRL, Chieti, Italy; ^6^ Department of Clinical Sciences, DISCO, Università Politecnica delle Marche, Ancona, Italy; ^7^ Center of Clinical Pathology and Innovative Therapy, National Institute INRCA-IRCCS, Ancona, Italy; ^8^ Section of Pathological Anatomy, Department of Biomedical Sciences and Public Health, Università Politecnica delle Marche, United Hospitals, Ancona, Italy

**Keywords:** bladder cancer, Trop-2, miR-125b, miRNA, immunohistochemistry

## Abstract

Human trophoblast cell surface antigen 2 (Trop-2) is a 40-kDa transmembrane glycoprotein that was first identified as a marker of human trophoblast cells. Trop-2 acts on cell proliferation, adhesion, and migration by activating a number of intracellular signalling pathways. Elevated Trop-2 expression has been demonstrated in several types of cancer and correlated with aggressiveness and poor prognosis. Since no data are available on Trop-2 in bladder cancer (BC), the purpose of the study was to determine its levels in tissue specimens from normal individuals and patients with BC at different stages. Moreover, since according to recent evidence Trop-2 is a miR-125b target, miR-125b expression was also assessed in tissue specimens. Finally, the effect of the Trop-2/miR-125b axis on the proliferation and migration of BC cells was evaluated *in vitro*.

The Trop-2/miR-125b axis was seen to be differentially expressed in normal urothelium, non-invasive BC and invasive BC tissue. Significant miR-125b down-regulation was associated with a significant increase in Trop-2 protein levels in BC tissue and correlated with disease severity. *In vitro* analysis confirmed the role of miR-125b in down-modulation of Trop-2 protein levels and showed that Trop-2/miR-125b axis affects cellular proliferation in bladder tissue.

In conclusion, our findings highlight a role for the Trop-2/miR-125b axis in BC progression and suggest Trop-2 and miR-125b as diagnostic/prognostic marker candidates as well as druggable targets for innovative therapeutic approaches.

## INTRODUCTION

As the second most common genitourinary malignancy, bladder cancer (BC) is a significant public health problem worldwide, with urothelial cell carcinoma (UC) accounting for nearly 90% of all primary BCs [1] and muscle-invasive BC (MIBC) for approximately 25 % of high mortality BCs. Clinical presentation is heterogeneous, and despite the availability of accurate histology-based classification systems it is difficult to predict prognosis and response to therapy. BC diagnosis is based on a combination of urine cytology, cystoscopy, and bladder biopsy. However, urine cytology is poorly sensitive to early, low-grade lesions, whereas cystoscopy is invasive, expensive, and only detects large lesions [2, 3].

Human trophoblast cell surface antigen 2 (Trop-2), a 40-kDa transmembrane glycoprotein encoded by the *TACSTD2* gene [4], has first been identified in human trophoblast and choriocarcinoma cell lines [5]. Its short intracytoplasmic tail has a key role in controlling several pathways that regulate cellular functions such as cell-cell adhesion, cell proliferation, and mobility [6, 7]. Trop-2 protein expression is often increased in a variety of epithelial cancers [8, 9]. A role as a marker of human prostate cancer stem cells has been proposed for it, particularly in cancer initiation and progression [10–12]. Trop-2 overexpression has been correlated with an aggressive malignant phenotype and poor prognosis [13–19]. The data reviewed above have made it an attractive diagnostic and prognostic marker candidate. Recently, Trop-2 is also being tested as a druggable target, since an anti-Trop-2 antibody-drug conjugate is being used to treat patients with several metastatic neoplasms, including triple-negative breast cancer and non-small-cell and small-cell lung cancer [20].

MicroRNAs (miRNAs) are short, non-coding RNAs that have attracted strong interest for their ability to affect the post-transcriptional stability of target mRNAs [21, 22]. Alteration of their expression has been described in several human diseases including cancer [23–29]. In particular, miR-125b is deregulated in a variety of malignancies, where it acts both as a tumour suppressor and as a promoter [30, 31]. MiR-125b is significantly down-modulated in BC bladder tissue compared with normal tissue [32]. A recent miRNA profiling and bioinformatic study has suggested that miR-125b-5p, along with *CDK4* and *CDK6* genes, may be involved in the BC pathway [33], and a subsequent investigation has associated it with MIBC progression and aggressiveness [34]. Moreover, according to recent evidence Trop-2 is a miR-125b target [35], but no data are available on the modulation of Trop-2/miR-125b in human BC. In this study, the levels of Trop-2 and miR-125b were investigated in normal bladder tissue and in urothelium from BC patients, to explore their potential as new diagnostic/prognostic biomarkers.

## RESULTS

### Tissue samples

A consecutive series of 40 samples was analysed. They were 9 specimens of healthy tissue, 21 specimens of non-invasive BC and 10 specimens of invasive BC collected from 40 subjects, of whom 31 (77.5%) were males (respectively n=6, n=18, and n=7). No significant association was detected between BC group and gender and there were no significant differences in age distribution between controls and cases.

### Trop-2 expression in normal and cancer tissue

**Figure 1:**
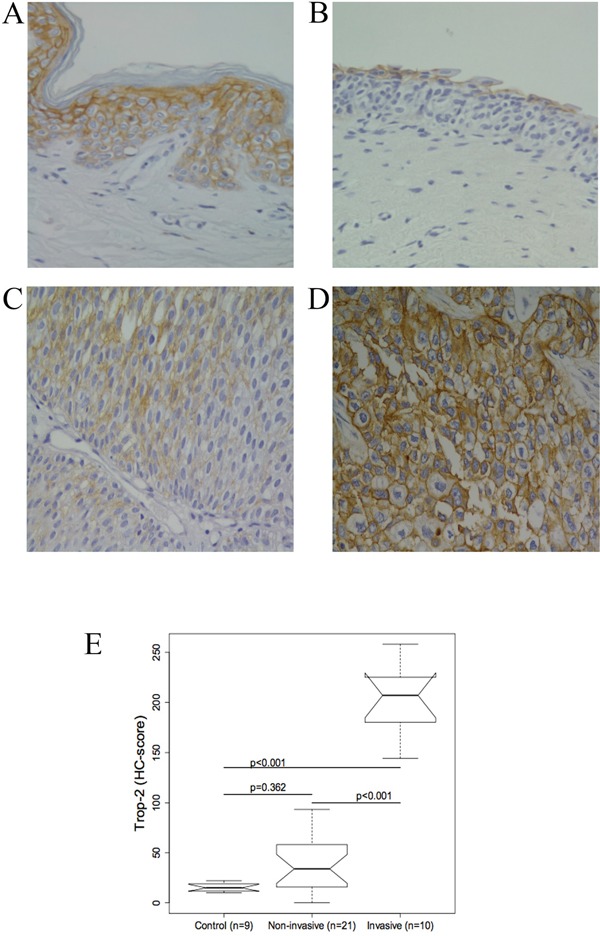
Representative immunohistochemical Trop-2 staining in normal bladder tissue and urothelium from patients with bladder cancer (BC) **(A)** Normal human skin was the positive control. **(B)** Normal bladder urothelium: Trop-2 expression in superficial urothelial cells. **(C)** Non-invasive BCs: cells are moderately stained. **(D)** Invasive BCs: strong Trop-2 expression. (original magnification, 40×) **(E)** Boxplot of the Trop-2 immunohistochemical score (HC score). Trop-2 protein staining was significantly different between invasive BCs and controls and between non-invasive and invasive BCs (all p<0.001). The difference between controls and non-invasive BCs was not significant (p = 0.362).

To determine whether Trop-2 protein levels were altered in human BC, Trop-2 immunostaining was performed in control and BC tissue. Normal human skin was used as a positive control (Figure 1A).

In normal tissue, Trop-2 staining was mainly confined to the superficial layer of the bladder urothelium, while stromal and endothelial cells were not stained. The immunostaining was strong in the superficial layer and very weak in the deeper layer (Figure 1B, 1E).

Different levels of Trop-2 were measured in different tumour stages. Compared with normal tissue Trop-2 levels were higher in non-invasive BC types (PUNLMP, and low- and high-grade papillary urothelial cancer) (Figure 1C, 1E), where staining of tumour cells ranged from negative to intermediate (Figure 1C). The invasive BCs showed Trop-2 protein overexpression that peaked in the cells invading the muscle (Figure 1D).

Trop-2 expression was significantly higher in invasive BCs compared both with normal bladder and with non-invasive BCs (both p ≤ 0.001), thus positively correlating with disease severity, whereas the difference between control and non-invasive BC tissue was not significant (Figure 1E).

### MiR-125b expression and correlation with Trop-2 levels in bladder tissue

**Figure 2:**
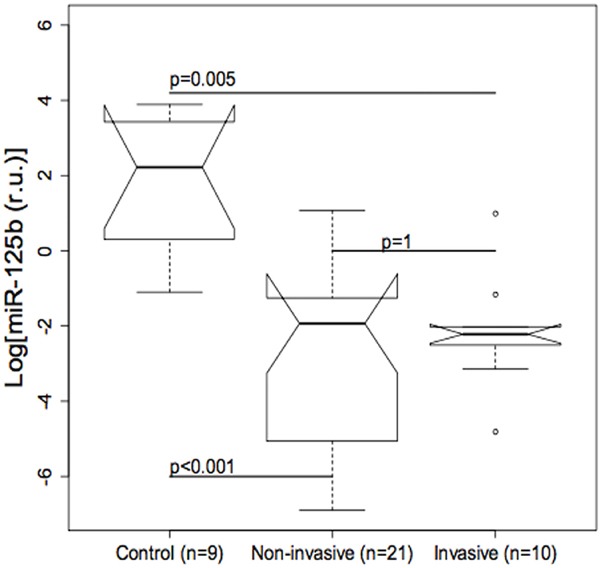
Boxplot of miR-125b values in bladder tumour and control tissue The difference between non-invasive (p<0.001) and invasive (p=0.005) urothelial bladder cancers (BCs) compared with controls is significant, whereas the difference between non-invasive and invasive BCs is not significant.

**Figure 3:**
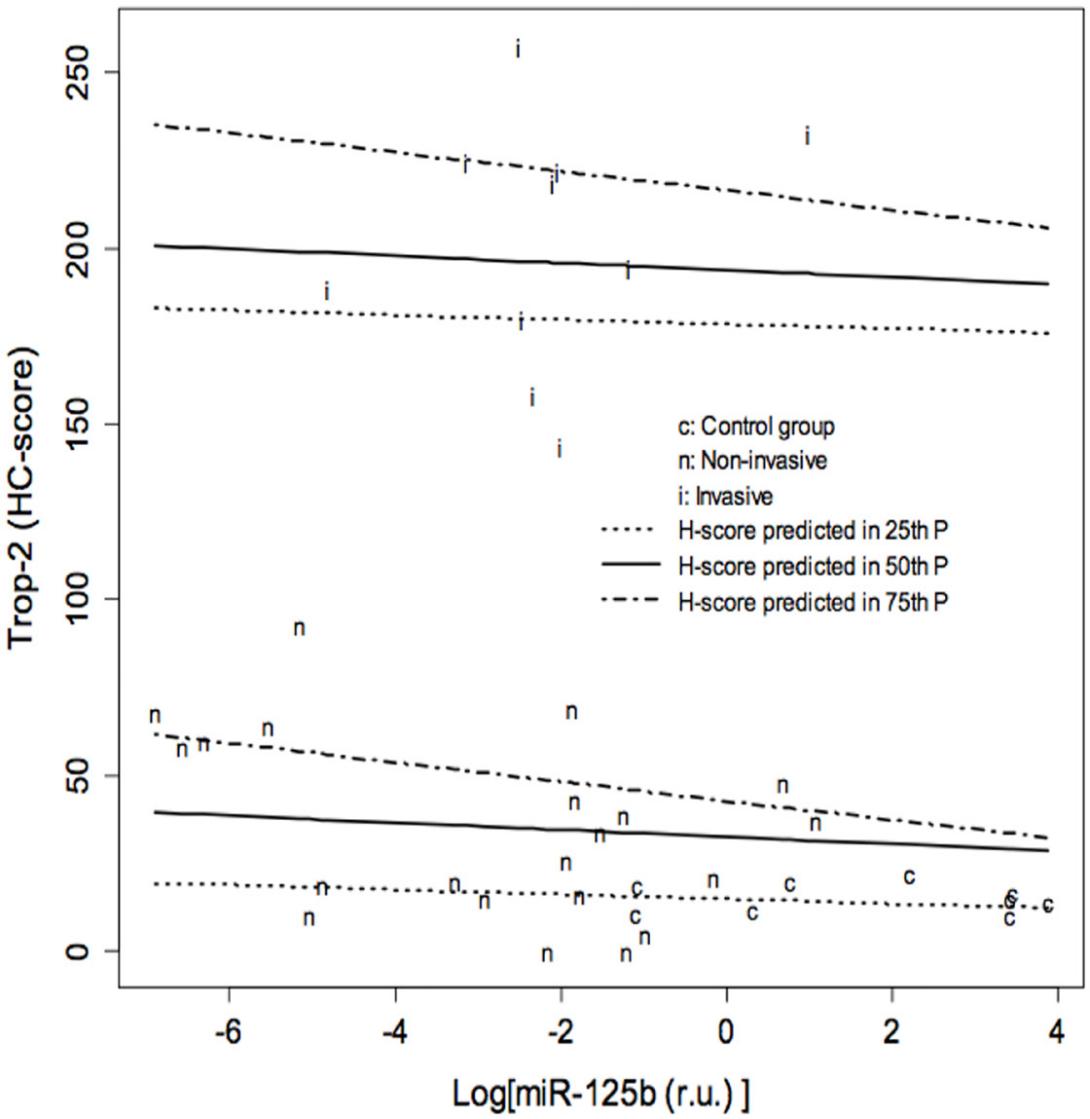
Results of quantile regression analysis of the effect of log (miR-125b) on the Trop-2 immunohistochemical score (HC score) in the three groups of specimens Results of quantile regression analysis for each quartile. The Trop-2 values of invasive bladder cancers (BC) are significantly higher compared to the control group in all three quartiles, whereas the values of non-invasive BCs are significantly higher only in the 3rd quartile. A significant negative association between log (miR-125b) and Trop-2 is found in the 3rd quartile. CI: Confidence Intervals. t= 0.25, t= 0.5, t=0.75: 25^th^, 50^th^ and 75^th^ percentiles of HC score distribution, respectively.

The possible role of miR-125b as a modulator of Trop-2 in urothelial BC was explored by measuring miR-125b levels in bladder tissue using real-time qPCR. MiR-125b expression was significantly lower in non-invasive (p ≤ 0.001) as well as invasive BCs (p=0.005) compared with control specimens, whereas the difference between invasive and non-invasive BCs was not significant (Figure 2). Quantile regression analysis showed significantly higher Trop-2 values in invasive BCs compared with the control group in all three quartiles, whereas Trop-2 values in the non-invasive BC group were significantly higher only in the 3^rd^ quartile. Moreover, a significant negative association between log (miR-125b) and Trop-2 was found in the 3^rd^ quartile (Figure 3, Table 1). The present findings show that miR-125b up-regulation checks Trop-2 expression.

**Table 1:** Results of quantile regression analysis of the effect of log (miR-125b) on the Trop-2 immunohistochemical score (HC score) in the three groups of specimens

	τ= 0.25		τ= 0.5		τ= 0.75	
	RegressionCoefficients	95% CI	RegressionCoefficients	95% CI	RegressionCoefficients	95% CI
Group: Non-invasive vs Control	2.64	-15.72; 15.51	14.55	-25.01; 25.55	18.22	0.03; 41.73
Group: Invasive vs Control	166	137; 193	176	131; 212	192	182; 214
Log [miR-125b (r.u.)]	-0.65	-6.48; 2.2	-1.01	-5.9; 1.77	-2.72	-4.82; -1.22

### MiR-125b expression and correlation with Trop-2 level in cell cultures and their role in proliferation and migration

**Figure 4:**
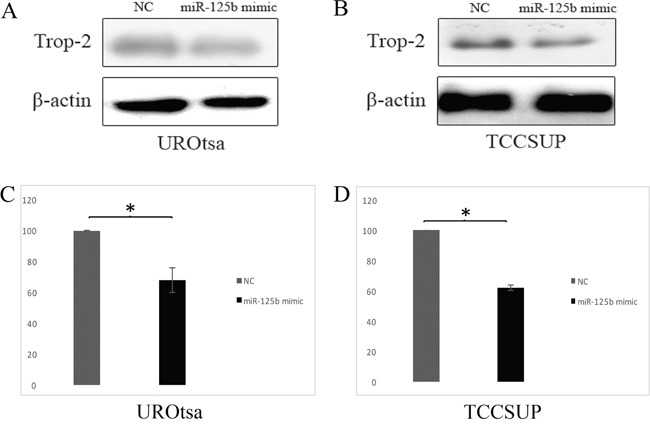
Effects of miR-125b on Trop-2 expression in transfected UROtsa and TCCSUP cells and negative control (NC) **(A)** Representative western blot of Trop-2 in UROtsa cells transfected with a miR-125b mimic and NC. **(B)** Representative western blot of Trop-2 in TCCSUP cells transfected with a miR-125b mimic and NC. **(C)** Densitometry graph of Trop-2 expression in UROtsa cells. **(D)** Densitometry graph of Trop-2 expression in TCCSUP cells. Trop-2 is significantly downregulated by the action of miR-125b both in UROtsa **(A, C)** and TCCSUP **(B, D)** cells. Data are expressed as percentage of Trop-2 expression in cells transfected with miR-125b compared to cells transfected with the NC (considered as 100%). P values are from paired samples t-test. *p≤0.01.

Transfection with a miR-125b mimic induced a very similar effect as endogenous miR-125b expression. The miR-125b overexpression induced by transient transfection was associated with a significant Trop-2 protein reduction in UROtsa (Figure 4A, 4C) and TCCSUP cells (Figure 4B, 4D).

**Figure 5:**
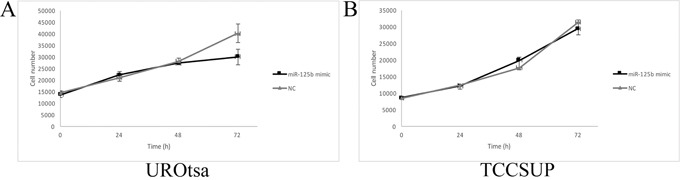
Cell growth curves of transfected UROtsa **(A)** and TCCSUP **(B)** cells. **(A)** In UROtsa cells growth was modulated by the miR-125b mimic 72 h after transfection compared to cells transfected with the negative control (NC). (n=5) p≤0.03 **(B)** In TCCSUP cells there were no differences between cells transfected with the miR-125b mimic and NC. (n=5) p≥0.05.

**Figure 6:**
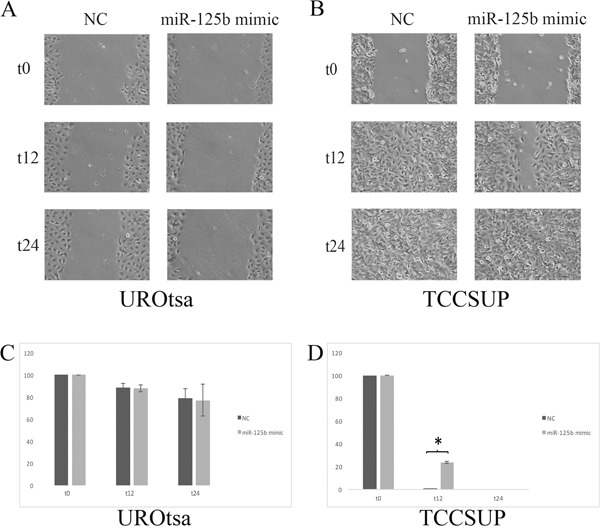
**(A)** Effect of transient transfection on UROtsa cell mobility assessed with the wound healing assay. **(B)** There are no significant differences. **(C)** Transient transfection of TCCSUP cells with miR-125b enhanced wound closure. Wound closure is complete at t24 hours. At t12 hours **(D)** there was a significant difference (*=p≤0.05) between TCCSUP cells transfected with the negative control (NC) and those transfected with the miR-125b mimic.

In UROtsa cells transfected with the miR-125b mimic, a significant reduction in the proliferation rate was measured between 48 h and 72 h compared with the cells transfected with the negative control (p ≤ 0.05, Figure 5A), whereas the growth rate of TCCSUP cells was not significantly affected (Figure 5B). In the wound healing assay, transfected UROtsa cells were not significantly different from control cells (Figure 6A, 6B), whereas transfected TCCSUP cells showed a significantly lower migration rate compared with control cells 12 h after incubation (Figure 6C, 6D); however, 24 h after incubation, they cells filled the wound in both conditions.

## DISCUSSION

Trop-2 protein participates in cellular process involved in tumour development and progression, such as proliferation and mobility [7–19]. Drugs targeting Trop-2 are a novel anti-cancer therapeutic strategy [20]. However, there are no data regarding Trop-2 in BC. This study shows for the first time that Trop-2 expression increases with increasing BC severity, showing a weak expression in normal bladder and a strong expression in invasive BC. Following the demonstration that Trop-2 mRNA is a target for miR-125b in head and neck squamous cell carcinoma [35], we decided to analyse the Trop-2/miR-125b axis in several types of BC. An inverse correlation was detected between Trop-2 and miR-125b expression, that was particularly strong in invasive BCs. These data suggest that Trop-2 is a target of miR-125b also in the bladder. To validate these findings, two different cell lines, UROtsa and TCCSUP cells, were employed in functional studies. Using transfection, we demonstrated that Trop-2 is a miR-125b target in both cell lines, which suggests that miR-125b plays a role in modulating Trop-2 protein expression in both normal bladder and cancer cell lines. Trop-2 expression decreased when cells were transfected with miR-125b, supporting the evidence obtained in bladder tissue. Moreover, since Trop-2 is crucially involved in cell proliferation and motility [7, 9, 12], we investigated the role of miR-125b in UROtsa and TCCSUP cell proliferation and motility by transfecting them with a miR-125b mimic. The results of this assay confirmed that the up-regulation of miR-125b and the consequent down-regulation of Trop-2 correlated with a reduced proliferation rate, suggesting that Trop-2 enhances urothelial cell proliferation. In addition, wound healing assay with transiently transfected cells showed that the miR-125b up-regulation and Trop-2 down-regulation inhibited cell migration suggesting that Trop-2 enhances bladder cell proliferation rate and motility. Interestingly, the cell proliferation and migration assays confirmed the immunohistochemical findings obtained in tissue sections, showing significantly increased Trop-2 levels and miR-125b down-regulation in the invasive BC types. The present data agree with the oncogenic role of Trop-2 proposed by Nakanishi and co-workers [35], reinforcing the notions that it plays a pivotal role in cancer pathogenesis and that miR-125b could be a modulator of this process. These findings are also consistent with a role for miR-125b in cancer development and progression, described in previous papers [36–42]. In particular, Zhao et al. [43] showed that overexpression of miR-125b significantly inhibited cell growth and suppressed migratory ability by Sphingosine Kinase 1 (SphK1) down-regulation in T24 bladder cancer cell line. Although we have used two different cell lines, the above reported data support our findings on miR-125b involvement in bladder cancer progression confirming the role of Trop-2 as oncogene. It is known that the majority of miRNAs does not act alone but in cluster and that each miRNA has more than one target. It is reasonable to think that the upregulation of miR-125b expression alters the balance between miRNAs/mRNA targets clusters thus resulting in a significant effect on a set of proteins. In conclusion, one of the most important novelty of our work is the identification and validation of a new target of miR-125b, like Trop2, in the context of BC, thus confirming an important role of both molecules in BC development.

Future studies should address the identification of other miRNAs involved in Trop-2 modulation in BC development and progression and evaluate the relevance of miRNAs as diagnostic/prognostic markers and as druggable targets for innovative therapeutic approaches.

## MATERIALS AND METHODS

### Tissue sample analysis

The procedure for this research project complies with the World Medical Association Declaration of Helsinki (World Medical Association. https://www.wma.net/policies-post/wma-declaration-of-helsinki-ethical-principles-for-medical-research-involving-human-subjects/. Accessed 25 Feb 2016.). All procedures were performed according to relevant national regulations and institutional guidelines. The study was approved by the Ethics committee on investigations involving human samples (Università Politecnica delle Marche, Italy). The study included 31 tissue specimens of UC (Table 2) and 9 specimens of normal urothelium. All specimens came from consecutive specimens collected from the Department of Urology (Università Politecnica delle Marche–United Hospitals, Ancona, Italy) and were processed and analysed at the Pathological Anatomy Section of the same institution. Patients’ clinical data were obtained from the records of the Pathological Anatomy Section. Tumour staging was according to the 2010 TNM classification system [44], whereas the BC histopathological classification was according to the 2016 World Health Organization [45]. All samples were fixed in 4 % neutral buffered formaldehyde solution for approximately 24 h before processing. Specimens were then routinely processed for paraffin embedding at temperatures not exceeding 56 °C. The diagnosis was made on sections stained with haematoxylin-eosin.

**Table 2:** Histological characteristics

Characteristics	No. of cases n=31
Histological grade	
PUNLMP	9
Papillary low-grade	6
Papillary high-grade	6
Invasive	10
pT category	
Ta	18
T1	3
T2b	3
T3a	1
T3b	4
T4a	2
pN (n=10)	
Nx	1
N0	1
N1	1
N2	7

### Immunohistochemical procedures

Immunohistochemical staining was performed in 4-μm-thick paraffin-embedded tissue sections. Sections were deparaffinized and rehydrated with xylene and a graded series of ethyl alcohols (from 100 % to 50 %) before incubation with 3 % hydrogen peroxide for 30 min to block endogenous peroxidase activity. Non-specific antibody binding was blocked with normal goat serum diluted 1:75 (30 min at room temperature). Afterwards, sections were incubated overnight at 4 °C with anti-Trop-2 mouse monoclonal antibody (no. SC-376181, Santa Cruz Biotechnology, Dallas, TX, USA) diluted 1:200 in phosphate buffered saline (PBS). After washing in PBS, the antigen was visualized by the streptavidin-biotin-peroxidase complex method (Vector Laboratories, Burlingame, CA, USA) using a biotinylated goat anti-mouse secondary antibody diluted 1:200. 3’, 3’- diaminobenzidine hydrochloride (Sigma-Aldrich, St Louis, MO, USA) was used as the chromogen. Sections were counterstained with Mayer's haematoxylin, dehydrated, and mounted in Eukitt solution (Kindler GmbH and Co., Freiburg, Germany). Negative controls were obtained by replacing the primary or secondary antibody with PBS. Additional negative controls included isotype-matched immunoglobulins (no. SC-2025, Santa Cruz Biotechnology). Normal human skin was used as a positive control for Trop-2 staining [8].

### Immunohistochemical scoring

Four fields per section were randomly chosen and examined at 40× magnification. Staining intensity was scored as 0, 1, 2, or 3 (respectively negative, weak, intermediate, and strong). We counted 250 cells/field (1000 cells/sample). The average percentage of positive cells was calculated for each BC type. An immunohistochemical score (HC) for each specimen was calculated by multiplying staining intensity by the percentage of positive cells {HC score = (% of weakly positive cells × 1) +(% of cells showing intermediate positivity × 2) +(% of strongly positive cells × 3)}. The HC scores therefore ranged from 0 (negative) to 300 (all cells displaying strong staining intensity) [46].

### MicroRNA extraction from bladder tissue and cell lines

The FFPE RNA/DNA Purification Kit (Norgen Biotek Corporation, Thorold, ON, Canada) was used for total mRNA extraction from 10-μm-thick formalin-fixed paraffin-embedded tissue sections according to the manufacturer's instructions.

For RNA extraction, cells seeded in 6-well plates were lysed with TRIzol reagent (Ambion, Life Technologies, Carlsbad, CA, USA); lysates were collected and RNA purified with Direct-zol RNA MiniPrep kit (Zymo Research Corp., Irvine, CA, USA) according to the manufacturer's recommendations. Total RNAs were stored at -80 °C until use.

### Quantitative real-time PCR

The expression of miR-125b and RNU48 (endogenous control) was measured by qPCR. Total RNA was converted to cDNA using the TaqMan microRNA reverse transcription kit and the TaqMan MicroRNA assay primers (all from Thermo Fisher, Monza, Italy) according to the manufacturer's instructions. The mixture was incubated at 16 °C for 30 min, at 42 °C for 30 min, and at 85 °C for 5 min.

Subsequently, quantitative real-time PCR was performed with TaqMan MicroRNA assay probes and the TaqMan Universal Master mix (Thermo Fisher) using reverse transcription product. The following thermal cycle protocol was performed using the Rotor-Gene Q real-time PCR cycler (Qiagen, Hilden, Germany): initial denaturation at 95 °C for 2 min followed by 40 cycles of 95 °C for 15 sec and 60 °C for 1 min.

Data were analysed with real-time PCR Rotor Gene Q Software 2 (Thermo Fisher). MiR-125b-fold changes were calculated by the delta Ct method (2^-ΔCt^) and its expression was determined using RNU48. All reactions were run in duplicate.

### Cell culture and miR-125b transfection in UROtsa and TCCSUP cells

UROtsa immortalized human urothelial cells (Rossi et al, Environ Health Perspect. 2001) and TCCSUP human bladder transitional carcinoma cells were cultured in Dulbecco's Modified Eagle's Medium (DMEM) supplemented with 10 % foetal bovine serum (FBS), 1% L-glutamine (all from Lonza, Basel, Switzerland) and 1 % penicillin/streptomycin (Sigma Aldrich, St Louis, MO, USA). Cells were grown at 37 °C in an atmosphere of 5 % CO_2_ and 95 % humidity. The medium was changed twice a week and cells were split 1:3 every 7 days, using a mixture of trypsin-EDTA (Lonza, Cologne, Germany). UROtsa cells were a kind gift from Scott H. Garrett (Department of Urology, West Virginia University at Morgantown, West Virginia, USA). TCCSUP cells were kindly provided by Axel Ullrich (Max-Planck Institute of Biochemistry, Department of Molecular Biology, Martinsried, Germany). All cell lines were free of mycoplasma contamination as routinely assessed by PCR analysis [47].

### Transient transfection

Transient miRNA transfection was performed using the X-tremeGENE 9 transfection reagent (Roche Applied Science, Mannheim, Germany) according to the manufacturer's instructions. Cells were seeded at a density of 2 × 10^4^/cm^2^ and transfected 12 h later with the mirVana miR-125b mimic or the negative control (Life Technologies, Carlsband, CA, USA). The miRNA mimic was used at the optimized concentration of 30 nM with the X-tremeGENE 9 transfection reagent (3:1 volume ratio). Transfected cells were incubated for 48 h before assaying. Untreated cells incubated with complete medium were used as an additional control. The level of miR-125b expression in both UROtsa and TCCSUP transfected cells was assessed by RT-PCR. UROtsa cells showed a very low basal expression level of miR-125b while TCCSUP cells showed an high basal expression level of miR-125b. Notably, the differences of miR125b increase after transfection were statistically significant in both cell lines (p<0.01 and p=0.039 respectively).

### Western blot analysis of Trop-2 from transfected UROtsa and TCCSUP cells

UROtsa and TCCSUP pellets were lysed in PBS 1 X, 0.1 % SDS, 1 % Nonidet-P40, 12 mM sodium deoxycholate, 1 mM sodium orthovanadate, 1 mM PMSF, and 1.7 μg/ml aprotinin. Samples were centrifuged at 14,000 rpm for 20 min at 4 °C. Protein supernatants were stored at -80 °C and quantified using the Bradford assay and a standard curve of bovine serum albumin (USB Corporation, Cleveland, OH, USA) concentrations ranging from 2 to 10 μg. Absorbance was read at 595 nm.

Equal amounts of protein in Laemmli sample buffer (4 % SDS, 10 % β-mercaptoethanol, 20 % glycerol, 0.004 % bromophenol blue, and 0.125 M Tris-HCl) were loaded and fractionated over a 15 % sodium dodecyl sulphate-polyacrylamide gel (SDS-PAGE) run at 110 V. The proteins were electrophoretically transferred to polyvinylidene fluoride membranes at 100 V for 1 h. Then membranes were incubated in 5 % milk (USB Corporation, Cleveland, OH, USA) in TBS/0.05 % Tween (TBS-T) for 1 h at room temperature to block non-specific protein binding. Blocked membranes were incubated overnight at 4 °C with primary antibodies: mouse anti-Trop-2, 1:250 (no. SC-376181) and goat anti-β-actin, 1:2000 (no. SC-1616), both from Santa Cruz Biotechnology. Membranes were washed 3 times with TBS-T and incubated with appropriate secondary antibodies conjugated with horseradish peroxidase; the anti-mouse reagent was diluted 1:1500 and the anti-goat reagent 1:3000. Detection of protein bands was performed with the SuperSignal West Pico Chemiluminescent Substrate (ECL) (Thermo Scientific, Waltham, MA, United States) as recommended by the manufacturer. Band intensity was quantified using Quantity One software (Bio-Rad Laboratories, Hercules, CA, USA). The relative quantity of Trop-2 was normalized against β-actin expression.

### Cell growth assay of cells transfected with miR-125b

For the cell growth assay, transfected cells were seeded at a density of 2 × 10^4^/cm^2^ in 96-well plates (5 replica wells/data point, 1 plate/time point). Transient transfections were performed as described above. Cells from each time point (0, 24, 48, 72 h) were fixed with 4 % paraformaldehyde for 40 min at room temperature. Cell growth was measured by staining with crystal violet (Sigma-Aldrich) [48]. Absorbance of acetic acid-released stain was quantified at 560 nm.

Cell numbers were calculated from standard reference curves of serially diluted cell samples.

### Wound healing assay of cells transfected with miR-125b

Wound healing assay was performed using transfectants. Confluent cell monolayers were scratched with a 200-μl sterile pipette tip and gap areas were monitored at 0, 12 h, and 24 h under an inverted microscope (Nikon, Minato, Tokyo, Japan). Images of the same fixed fields were obtained at every time point. Gap areas were quantified using ImageJ software (https://imagej.nih.gov/ij/).

### Statistical analysis

Due to the small sample size and the non-normal distribution as evaluated with the Shapiro test, the expression of Trop-2 and miR-125b in BC and control tissue specimens was tested with non-parametric approaches. MiR-125b levels were analysed after log transformation. Median and interquartile range (IQR) were used as a measure of centrality and variability, respectively, and graphically represented as boxplots. The Kruskal-Wallis test was applied to between-group comparisons. Quantile regression [49] was performed using the HC score as the dependent variable and BC groups and log (miR-125b) as explanatory factors. Quantile regression allowed estimating quantile-specific effects by describing the impact of exposure not only on the centre but also on each region of interest of the outcome distribution (e.g. distribution tails). Three parts of the HC score distribution were considered in the present analysis: 25^th^, 50^th^ and 75^th^ percentiles. Results were provided as regression coefficient estimates and 95 % confidence intervals (95% CI): if the 95 % CI did not include zero value, the regression coefficients were considered significantly different from 0. The R statistical program was used for the analyses and a probability of 0.05 was set as the threshold for statistical significance.

Data from cell experiments were expressed as the mean ± standard deviation (SD). The differences between the groups were analysed with Student's t-test when two groups were compared and p-value of <0.05 was considered to be statistically significant.
